# KLF5 inhibits STAT3 activity and tumor metastasis in prostate cancer by suppressing IGF1 transcription cooperatively with HDAC1

**DOI:** 10.1038/s41419-020-2671-1

**Published:** 2020-06-16

**Authors:** Jian-Bin Ma, Ji-Yu Bai, Hai-Bao Zhang, Jing Jia, Qi Shi, Chao Yang, Xinyang Wang, Dalin He, Peng Guo

**Affiliations:** 1 Department of Urology, The First Affiliated Hospital of Xi’an Jiaotong University, Xi’an, Shaanxi China; 2 Key laboratory for Tumor Precision Medicine of Shaanxi Province, Xi’an, Shaanxi China; 3 Oncology Research Lab, Key Laboratory of Environment and Genes Related to Diseases, Ministry of Education, Xi’an, Shaanxi China

**Keywords:** Metastasis, Prostate cancer

## Abstract

KLF5 is frequently deleted and downregulated in prostate cancer, and recently it has been reported that KLF5 loss is enriched in the aggressive branches of prostate cancer evolution. However, why KLF5 loss is associated with prostate cancer aggressiveness is still not clear. Herein, we analyzed KLF5 expression in TCGA and GEO database, as well as prostate cancer tissue microarray, and found that KLF5 expression significantly decreased in prostate cancer accompanying with tumor progression; moreover, KLF5 downregulation was associated with shorter survival of patients. Interestingly, we also found that KLF5 expression was obviously lower in prostate cancer metastases than in localized tissues, indicating that KLF5 downregulation is associated with prostate cancer invasion and metastasis. To assess this effect of KLF5, we knocked down KLF5 in prostate cancer cells and found that KLF5 knockdown promoted invasive ability of prostate cancer cells in vitro and in vivo. Moreover, we found that KLF5 downregulation enhanced the expression of IGF1 and STAT3 phosphorylation, while block of IGF1 with antibody decreased the enhancement of STAT3 activity and prostate cancer cell invasive ability by KLF5 knockdown, indicating that KLF5 inhibits prostate cancer invasion through suppressing IGF1/STAT3 pathway. Mechanistically, we found that KLF5 interacted with deacetylase HDAC1 and KLF5 is necessary for the binding of HDAC1 on *IGF1* promoter to suppress IGF1 transcription. Taken together, our results indicate that KLF5 could be an important suppressor of prostate cancer invasion and metastasis, because KLF5 could suppress the transcription of IGF1, a tumor cell autocrine cytokine, and its downstream cell signaling to inhibit cell invasive ability, and reveal a novel mechanism for STAT3 activation in prostate cancer. These findings may provide evidence for the precision medicine in prostate cancer.

## Introduction

Prostate cancer (PCa) is the most commonly diagnosed cancer and the second leading cause of cancer-related mortality in males in the USA^1^. Although a majority of patients with localized tumor responds to the initial treatment, nearly 20–30% of treated patients inevitably progress to castration-resistant PCa followed by cancer metastasis^2^, leading to poor prognosis and mortality^3^. However, only a few treatments are effective in metastatic PCa so far. Thus, there is an urgent need to develop novel diagnostic and therapeutic approaches based on the biological and molecular mechanisms underlying PCa metastatic spread.

Krüpple-like transcription factor 5 (KLF5/IKLF/BTEB2), which is a zinc-finger transcription factor, has various functions in different cellular processes, including proliferation, apoptosis, invasion, and differentiation^4,5^. KLF5 binds specifically to the CA-box and GC-rich regions on target promoters and can recruit other transcription factors and epigenetic enzymes, such as ERβ, SMAD4, p300, and HDAC3, into the transcriptional complex to activate or repress the target gene transcription^6–8^. Frequent deletion of the KLF5 gene was reported in human PCa^9^, and protein degradation and promoter DNA methylation also led to KLF5 downregulation in PCa^10^. Conditional *Klf5* deletion in mouse prostate epithelial cells promoted *Pten* deletion and initiated tumorigenesis^11^, further suggesting that KLF5 may function as a tumor suppressor in PCa. However, the association between KLF5 expression and the clinical features of PCa, and whether KLF5 regulates the invasiveness of PCa cells remain to be elucidated.

STAT3 activation plays an important role in PCa progression^12–14^. Most PCa metastases were positive for p-STAT3 staining and STAT3 inhibitor galiellalactone effectively decreased metastatic tumor spread in a mouse model of PCa^15^, indicating that STAT3 activation may be a crucial promotor in PCa invasion and metastasis. STAT3 can be activated by various cytokines, such as IL-6, CXCL-5, and COX2/PGE2, from PCa cells and the tumor microenvironment^16–18^. However, the activation of STAT3 in PCa metastasis is complex, and other cytokines may play important roles in this process, depending on the context. Since modulating STAT3 activity is a potential approach to treat PCa, a molecular understanding of the underlying mechanism(s) of STAT3 activation in PCa would provide evidence for developing precision medicine of PCa treatment.

In the present study, we analyzed the association between KLF5 expression and the clinical characteristics of PCa and determined whether KLF5 regulates the invasiveness of PCa cells. We further investigated the mechanism of KLF5 inhibition of the invasive ability of PCa cells by suppressing transcription of IGF1 and decreasing the activity of the IGF1/p-STAT3 signaling pathway. In summary, we found that KLF5 deletion/downregulation in PCa could promote tumor invasion and metastasis through modulating the cytokine IGF1, expressed by tumor cells, and the subsequent cell signaling.

## Materials and methods

### Cell culture and reagents

Human PCa cell lines 22RV1, PC-3, and DU145 were purchased from the American Type Culture Collection (Manassas, VA, USA). C4-2 cell line was a gift from Dr. Jer-Tsong Hsieh at the University of Texas Southwestern Medical Center. All cell lines were cultured in RPMI-1640 medium supplemented with 10% fetal bovine serum at 37 °C aired with 5% CO_2_. STAT3 inhibitor niclosamide (dissolved in DMF) was purchased from Selleckchem (Houston, TX, USA). All reagents were reconstituted and stored following the protocol.

### Plasmid and siRNA transfection, lentiviral infection

KLF5 knockdown lentivirus and scramble control were purchased from GeneCopoeia (Guangzhou, China). The 22RV1 cells were transfected with KLF5-overexpressing plasmid (HA-KLF5)^19^ with Lipofectamine® 3000 Reagent and P3000^TM^ Reagent Invitrogen (Thermo Fisher Scientific, Inc., Waltham, MA, USA) following the manufacturer’s instructions. IGF1 was knocked down by si-IGF1 (RIBOBIO, Guangzhou, China). For exogenous co-immunoprecipitation assay, pCMV3-HDAC1-Flag and HA-KLF5 plasmids were used to transiently transfect 293T cells for 48 h with lipofectamine® 3000 Reagent and P3000^TM^ Reagent. For dual-luciferase reporter assays, the plasmids of pGL3-Basic, pGL3-IGF1#1, pGL3-IGF1#2, and pGL3-IGF1#3 were used to transfect transiently shNC and shKLF5 sublines of 22RV1 and PC-3 cells for 48 h with Lipofectamine® 3000 Reagent and P3000^TM^ Reagent. All plasmids, siRNAs, and lentivirus transfection were performed following the manufacturer’s protocols.

### Cell invasion assay

Cell invasion was tested by the Boyden chamber assay, and details of the procedure are described in Supplementary materials and methods.

### Western blotting analysis

The procedure was described previously^8^. The following antibodies were used: KLF5 (ab24331), MMP9 (ab38898), and IGF1 (ab9572) (Abcam, Cambridge, UK), p-STAT3 (Tyr705) (#9145), STAT3 (#9139), HDAC1 (#5356), and AR (#5153) (Cell Signaling Technology, Beverly, MA, USA), MMP2 (10373-2-AP) (Proteintech, Rosemont, IL, USA), and β-actin (ComWin Biotech Co. Beijing, China).

### Tissue chip and immunohistochemistry (IHC) assays

To explore the expression of KLF5 in PCa tissues and adjacent tissues, we purchased a PCa tissue chip (Catalog No. HProA 180PG04) from Outdo Biotech (Shanghai, China) containing 90 samples of PCa tissues and 90 samples of adjacent tissues. Briefly, tissue chip was deparaffinized, rehydrated, followed by 5 min 121 °C antigen retrieval, 30 min of endogenous enzyme block, 1 h of 5% BSA block. Subsequently, the chip was incubated with the KLF5 primary antibody (Abcam, Cambridge, UK) (diluted at 1:200) overnight at 4 °C. Next, the tissue chip was incubated with EnVision-HRP secondary antibody for 1 h and the signals were detected by diaminobenzidine followed by hematoxylin counterstaining. Stained signals were photographed using an Olympus BX51 microscope (Olympus, Tokyo, Japan). Of note, the result was assessed according to the intensity of staining (0, 1+, 2+, and 3+) and the percentage of positive cells (0 (0%), 1 (1–25%), 2 (26–50%), 3 (51–75%), and 4 (76–100%)) by two experienced pathologists. Finally, the final staining score was calculated and analyzed.

### Xenograft tumor model and in vivo metastasis analysis

Twelve male athymic nude mice at 4 weeks of age were used according to the protocols approved by the ethical committee of Xi’an Jiaotong University (Permission Number: SCXK2018-0155, 5 March 2018). Briefly, male nude mice were injected i.v. via the tail vein with 5 × 10^6^ 22RV1 sublines (NC and shKLF5). Eight weeks later, nude mice were sacrificed, and the metastases were examined by H&E staining. The KLF5 (1:200) and p-STAT3 (1:100) expression levels in lung metastases were detected by IHC assay according to the protocol described above.

### Chromatin immunoprecipitation (ChIP) assay

We performed CHIP assays using Magna CHIP^TM^ A/G Kit from MERCK (Darmstadt, Germany) following the manufacturer’s protocol. The DNA/protein mixture was prepared from wild-type and shKLF5 subtypes of 22RV1 and PC-3 cells, and antibodies against KLF5 (Abcam) and HDAC1 (Cell Signaling Technology) were used to precipitate protein/DNA complex. The results were analyzed by the %input method.

### Dual-luciferase activity assay

The *IGF1* promoter report plasmids pGL3-IGF1#1, pGL3-IGF1#2, and pGL3-IGF1#3, in which −1895 to +105 bp, −1595 to +105 bp, and −1115 to +105 bp, respectively, of its promoter region (NG_011713.1) were inserted into the pGL3-basic plasmid, were constructed by Genechem Company (Shanghai, China) and validated by sequencing. TK promoter Renilla luciferase plasmid and *IGF1* promoter report plasmids were co-transfected into 22RV1 and PC-3 cells (shNC or shKLF5) at the ratio of 1:20. After 48 h, the luciferase assay was carried out using the Dual-Luciferase Assay kit (Promega, Madison, WI, USA) following the manufacturer’s instructions.

### Statistical analysis

GraphPad Prism version 5.0 software (GraphPad, San Diego, CA, USA) was used for analyzing differences between two groups (Student’s *t* test). *P* < 0.05 was regarded as the threshold value for statistical significance. All error bars in graphical data represent mean ± SD.

## Results

### Expression of KLF5 is decreased during prostate cancer progression, and its downregulation is associated with shorter survival of patients

**Fig. 1:**
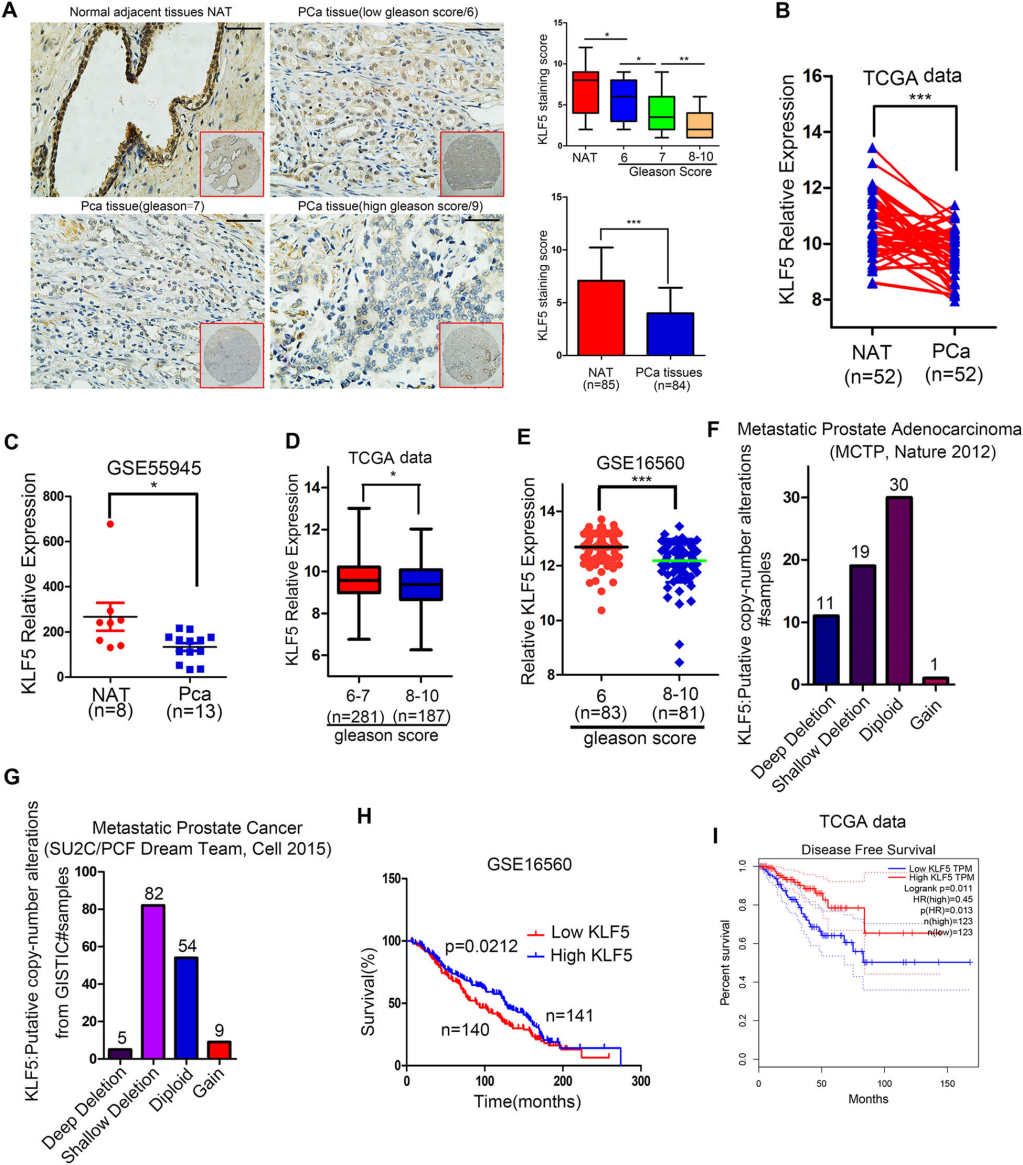
Decreased expression of KLF5 in PCa progression is associated with shorter survival of patients. **a** Representative picture of KLF5 protein expression in prostate cancer tissue chip detected by IHC and quantification of KLF5 protein in prostate cancer. Scale bar = 100 μm. **b**, **c** KLF5 mRNA expression in prostate cancer tissues and adjacent tissues from TCGA and GEO (GSE55945) databases. **d**, **e** KLF5 mRNA expression in prostate cancer tissues with different Gleason score from TCGA and GEO (GSE16560) databases. **f**, **g** Analysis of KLF5 gene putative copy-number alterations in metastases of prostate cancer from (MCTP) and (SU2C/PCF Dream Team) in cBioportal website. **h** Association between overall survival of prostate cancer patients and KLF5 mRNA expression from the GEO database (GSE16560). **i** Association between disease-free survival of prostate cancer patients and KLF5 mRNA expression from the TCGA database. *p < 0.05; **p < 0.01; ***p < 0.001.

To investigate the expression pattern of KLF5 in PCa tissues, we performed immunohistochemistry (IHC) analysis on a PCa tissue chip and observed downregulation of the KLF5 protein in PCa tissues compared with adjacent prostate tissues. Moreover, KLF5 protein was downregulated significantly in high Gleason score 8–10, a vital parameter to evaluate PCa progression, compared with low Gleason score 6 and 7 PCa tissues (Fig. 1a). To further explore the KLF5 mRNA expression level in PCa, we analyzed TCGA and GEO databases and found that expression of KLF5 mRNA was lower in PCa tissues compared with adjacent prostate tissues in the TCGA cohort and GEO series GSE55945 (Fig. 1b, c). Consistent with the KLF5 protein expression pattern in Fig. 1a, we found that KLF5 mRNA expression decreased in high Gleason score 8–10 PCa tissues compared with low Gleason score 6–7 tissues (Fig. 1d, e). It has been reported that *KLF5* gene is frequently deleted in PCa. Therefore, to further explore KLF5 deletion in PCa metastases, we analyzed data from two studies^20,21^. We detected deep and shallow deletion of the KLF5 gene in 11/61 and 19/61 metastatic tissues, respectively (Fig. 1f) and deletion of KLF5 gene occurred in 87/150 metastatic PCa samples (Fig. 1g). We also found that KLF5 expression was correlated with the overall survival of PCa patients (Fig. 1h) and disease-free survival (Fig. 1i) in the TCGA and GSE16560 cohorts.

In summary, our results indicated that KLF5 expression decreased during PCa progression and was associated with shorter survival of patients.

### KLF5 Knockdown promotes invasion of prostate cancer cells in vivo and in vitro

**Fig. 2:**
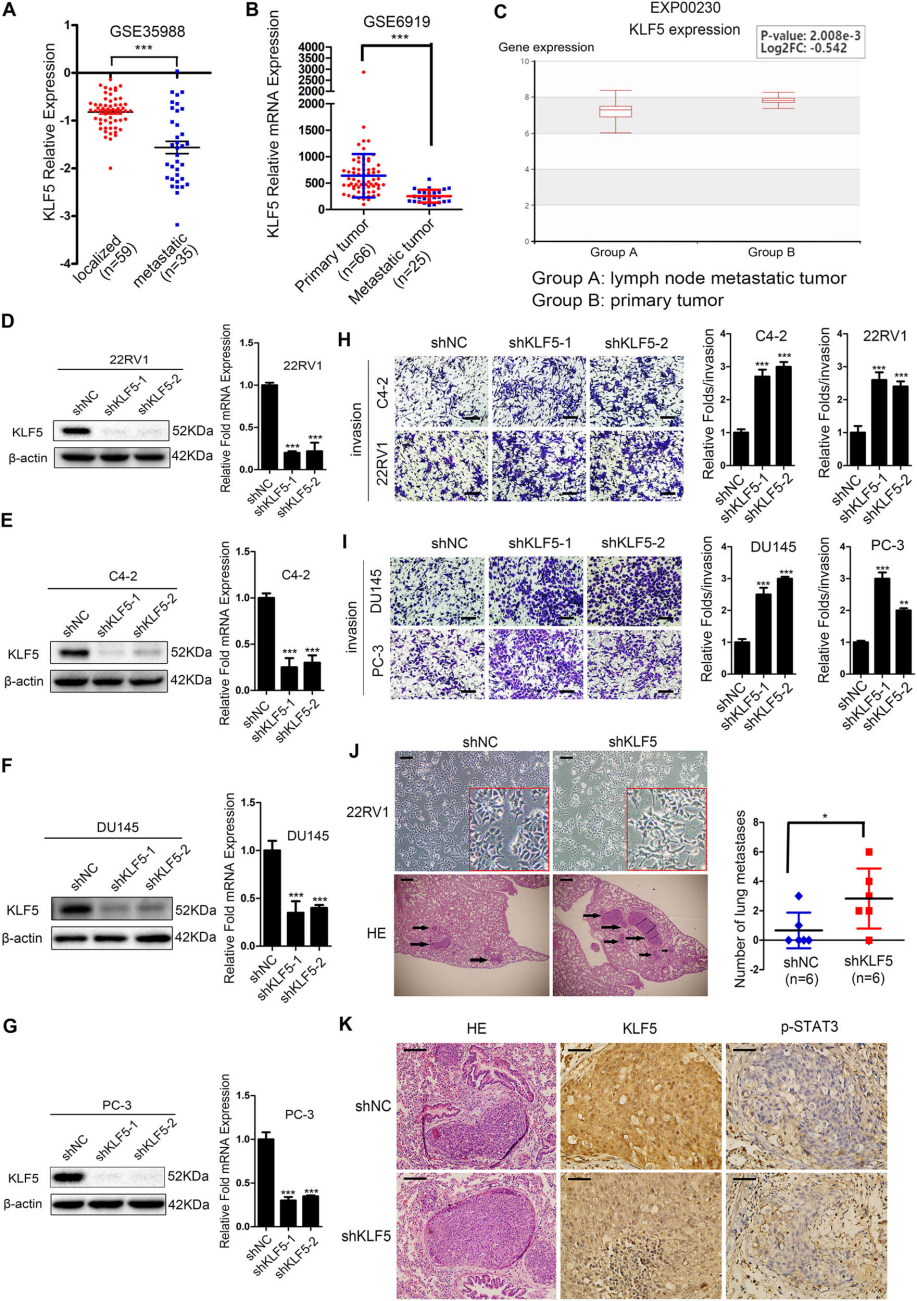
Knockdown of KLF5 promotes invasion of prostate cancer cells in vitro and in vivo. **a**, **b** KLF5 mRNA expression in localized and metastatic tissues of prostate cancer from GEO databases GSE35988 and GSE6919. **c** KLF5 mRNA expression was lower in lymph node metastatic tumors than in primary prostate tumors as per EXP00230 of the HCMDB website. **d**–**g** Real-time quantitative PCR and western blotting of KLF5 mRNA and protein levels in C4-2, 22RV1, DU145, and PC-3 cells transfected with KLF5 shRNA (shKLF5) or negative control (NC). 18S and β-actin were used as loading controls. **h**, **i** Representative Transwell data and quantification analysis of invasion assays in C4-2, 22RV1, DU145, and PC-3 cells transfected with KLF5 shRNA or shNC. Scale bar = 100 μm. The data are from at least three independent experiments expressed as the mean ± SD. *p < 0.05; **p < 0.01; ***p < 0.001. **J** Representative microscopic images of the morphology of 22RV1 cells after KLF5 knockdown (upper panel), and representative histological (lower panel) and quantification analyses of nude mice lung metastatic foci in 22RV1/NC and 22RV1/shKLF5 groups. Scale bar = 100 μm. **k** Immunohistochemistry staining of KLF5 and p-STAT3 in nude mice lung metastatic tissues from 22RV1/NC and 22RV1/shKLF5 groups. Scale bar = 100 μm.

**Fig. 3:**
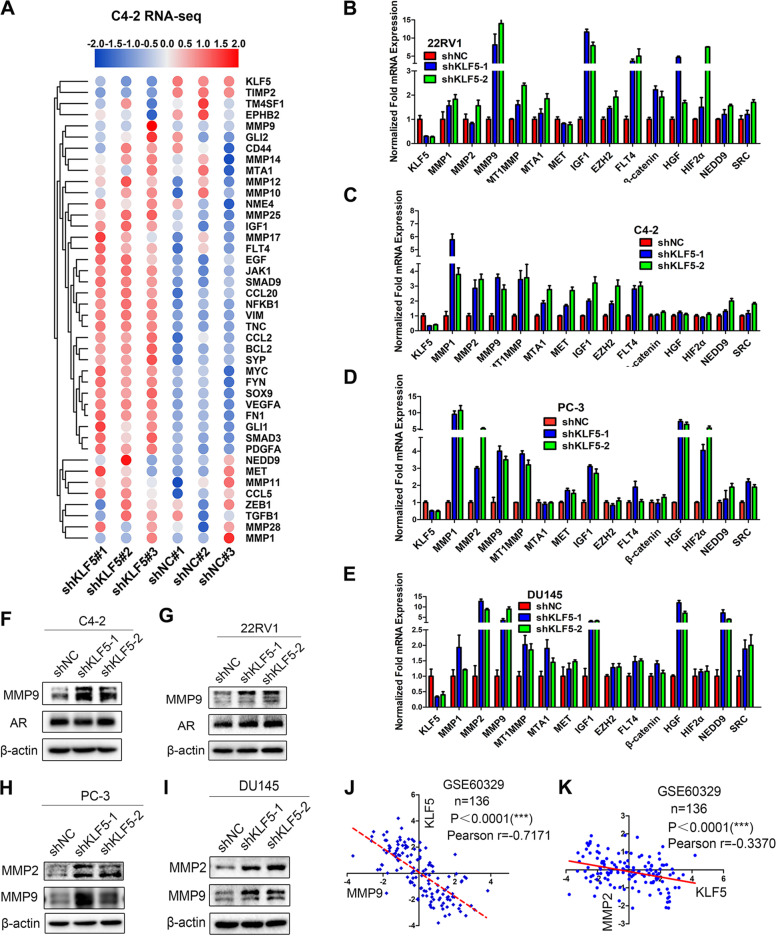
Knockdown of KLF5 up-regulates invasion-related markers in prostate cancer cells. **a** Heatmap shows cells invasion-related genes regulated by KLF5 knockdown in C4-2 sublines (C4-2-NC/C4-2-shKLF5) detected by high throughput sequencing (RNA-seq). **b**–**e** Effect of KLF5 knockdown on the expression of 14 invasion-associated genes in C4-2, 22RV1, PC-3, and DU145 cells, as detected by RT-qPCR. 18S was used as an internal loading control. **f**, **g** Western blotting of AR and MMP9 in C4-2 and 22RV1 AR-positive cell lines transfected with shKLF5 or shNC. **h**, **i** Western blotting of MMP2 and MMP9 in DU145 and PC-3 in AR-negative cell lines transfected with shKLF5 or shNC. **j** Analysis of linear correlation between KLF5 and MMP9 from GSE60329. **k** Analysis of linear correlation between KLF5 and MMP2 from GSE60329.

The deletion of the KLF5 gene contributes to its low expression in PCa metastatic tissues. We detected lower KLF5 mRNA expression in metastatic tumor tissues than in localized tumor tissues of PCa as evidenced by the analysis from GSE36988 (Fig. 2a), GSE6919 (Fig. 2b), and EXP00230 (Fig. 3c), indicating that KLF5 was downregulated in metastatic loci of PCa. To assess the effect of KLF5 downregulation on PCa invasion, we knocked down KLF5 in C4-2, DU145, 22RV1, and PC-3 PCa cells, successfully established stable sublines (Fig. 2d–g). Indeed, knockdown of KLF5 enhanced the invasiveness of C4-2, DU145, 22RV1, and PC-3 sublines as detected by invasion Transwell assay (Fig. 2h, i).

To further verify the tumor metastasis-suppressive role of KLF5 in PCa in vivo, we established metastasis mouse models using the tail-vein injection of 22RV1 sublines (22RV1-NC/22RV1-shKLF5). Histological analysis showed that nude mice receiving the 22RV1/shKLF5 subline formed a higher number of lung metastatic foci compared with 22RV1/NC control group (Fig. 2j). Also, by performing the IHC assay, we found downregulation of KLF5 in lung metastatic foci formed by 22RV1/shKLF5, while p-STAT3 was upregulated, indicating that KLF5 might regulate p-STAT3 expression in PCa. (Fig. 2k).

Taken together, our data indicated that KLF5 loss in PCa could promote the invasive ability of PCa cells in vitro and in vivo.

### KLF5 knockdown up-regulates invasion-related markers in prostate cancer cells

Since the loss of KLF5 increased the invasive ability of PCa cells, we next investigated the underlying mechanism of regulation of its target genes. Firstly, we detected how KLF5 downregulation changed gene expression profiling by high throughput RNA-sequencing in C4-2 sublines (C4-2-NC/C4-2-shKLF5) (Fig. 3a) and found that KLF5 knockdown upregulated a series of invasion-related genes, including IGF1 and MMPs family members. Interestingly, according to analysis on GSE58719, klf5 loss in mouse prostates also significantly upregulated invasion-related genes (Fig. S1A). Next, we examined the mRNA expression of 14 invasion-related genes in PCa cells and found a significant upregulation of IGF1 mRNA in all four cell lines when KLF5 was knocked down (Fig. 3b–e), suggesting that IGF1 was an essential target gene of KLF5 in PCa. This was supported by the finding that KLF5 overexpression suppressed of IGF1 mRNA level in DU145 cells (Fig. S1B). On the other hand, MMP9 protein level expression was upregulated in C4-2 and 22RV1 cells when KLF5 was knocked down (Fig. 3f, g). Different from AR-positive cell lines, KLF5 knockdown in PC-3 and DU145 cells increased protein levels of both MMP9 and MMP2 compared with the control group (Fig. 3h, i). Consistently, we found that KLF5 was negatively correlated with MMP9 and MMP2 at the mRNA level in GSE60329 from the GEO database (Fig. 3j, k).

In summary, these results indicated that KLF5 knockdown could significantly upregulate a series of critical invasion-related genes, further confirming that KLF5 downregulation could promote PCa cell invasion.

### KLF5 downregulation activates IGF1/p-STAT3 pathway in PCa promoting cell invasion

**Fig. 4:**
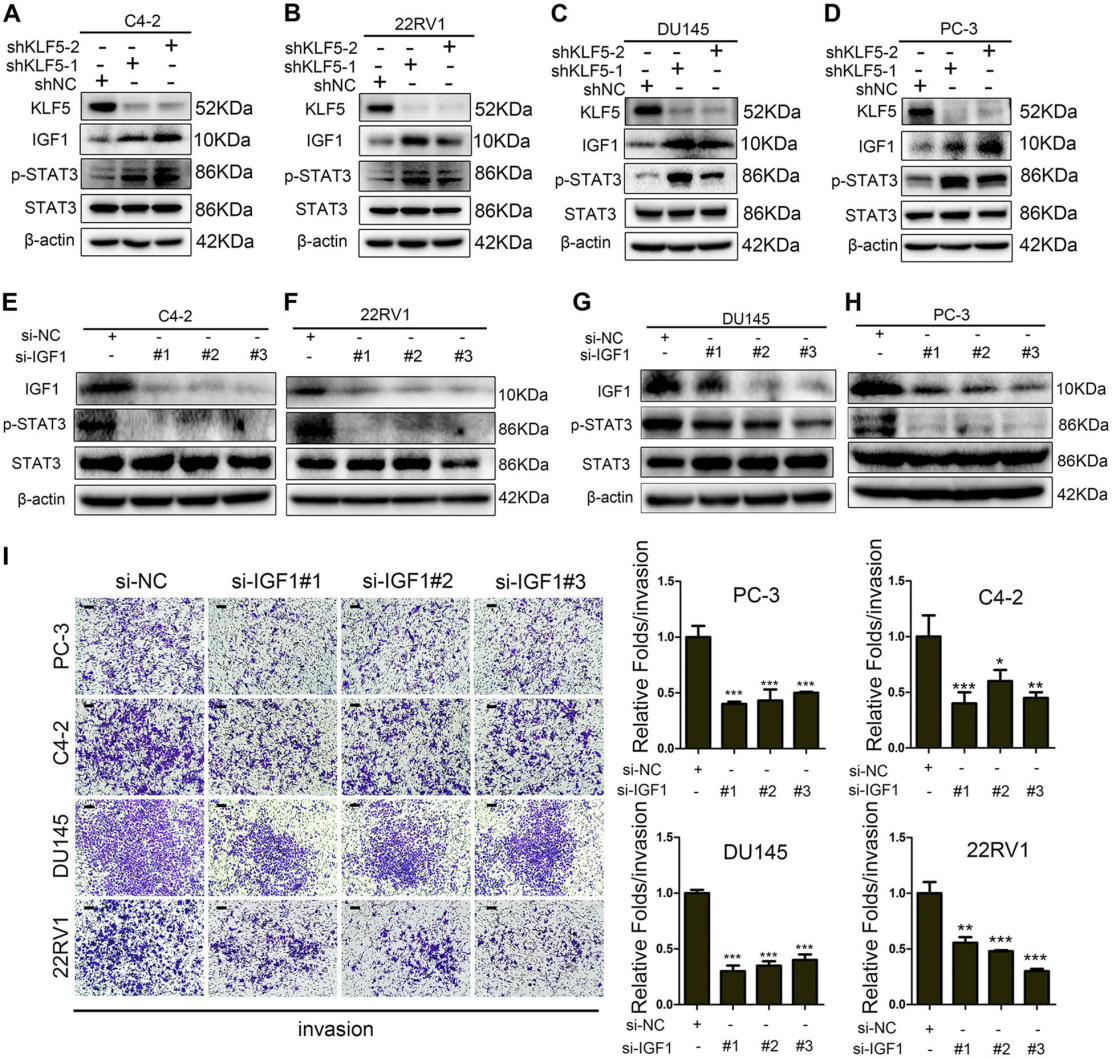
Downregulation of KLF5 activates the IGF1/STAT3 pathway and promotes cell invasion. **a**–**d** Western blotting analysis of KLF5, IGF1, p-STAT3, and STAT3 in C4-2, 22RV1, DU145, and PC-3 cells transfected with shKLF5 or shNC. **e**–**h** Western blotting analysis of IGF1, p-STAT3, and STAT3 expression in C4-2, 22RV1, DU145, and PC-3 cells transfected with si-IGF1 (si-IGF1#1, si-IGF1#2, si-IGF1#3) to knockdown IGF1. **i** Representative Transwell photographs and quantification analysis of invasion assays in C4-2, 22RV1, DU145, and PC-3 cells transfected for 48 h with si-IGF1 (si-IGF1#1, si-IGF1#2, si-IGF1#3). Scale bar = 100 μm; *p < 0.05; **p < 0.01; ***p < 0.001.

As shown in Fig. 3, knockdown of KLF5 upregulated IGF1 at mRNA level. Given that excessive activation of the STAT3 pathway occurs in PCa metastatic tissues^15^ and IGF1/IGF1R can activate STAT3 and its downstream target genes, such as MMP9^22^, we hypothesized that KLF5 might regulate STAT3 activity through IGF1. Western blotting revealed that KLF5 knockdown could promote IGF1 protein expression in various PCa cell lines (Fig. 4a–d), and p-STAT3 was also increased. To verify that IGF1 induced the activation of STAT3 in PCa cells, we knocked down IGF1 with si-IGF1 in C4-2, 22RV1, DU145, and PC-3 cells and found p-STAT3 expression to be reduced (Fig. 4e–h). To explore whether IGF1 promoted the invasive ability of PCa cells, we performed Transwell invasion assay and observed that downregulation of IGF1 significantly inhibited invasion of PCa cells (Fig. 4i).

Overall, these results indicated that downregulation of KLF5 activated the IGF1/STAT3 pathway, which in turn, promoted the invasive ability of PCa cells.

### KLF5 downregulation promotes prostate cancer cell invasion via activating IGF1/STAT3 pathway

**Fig. 5:**
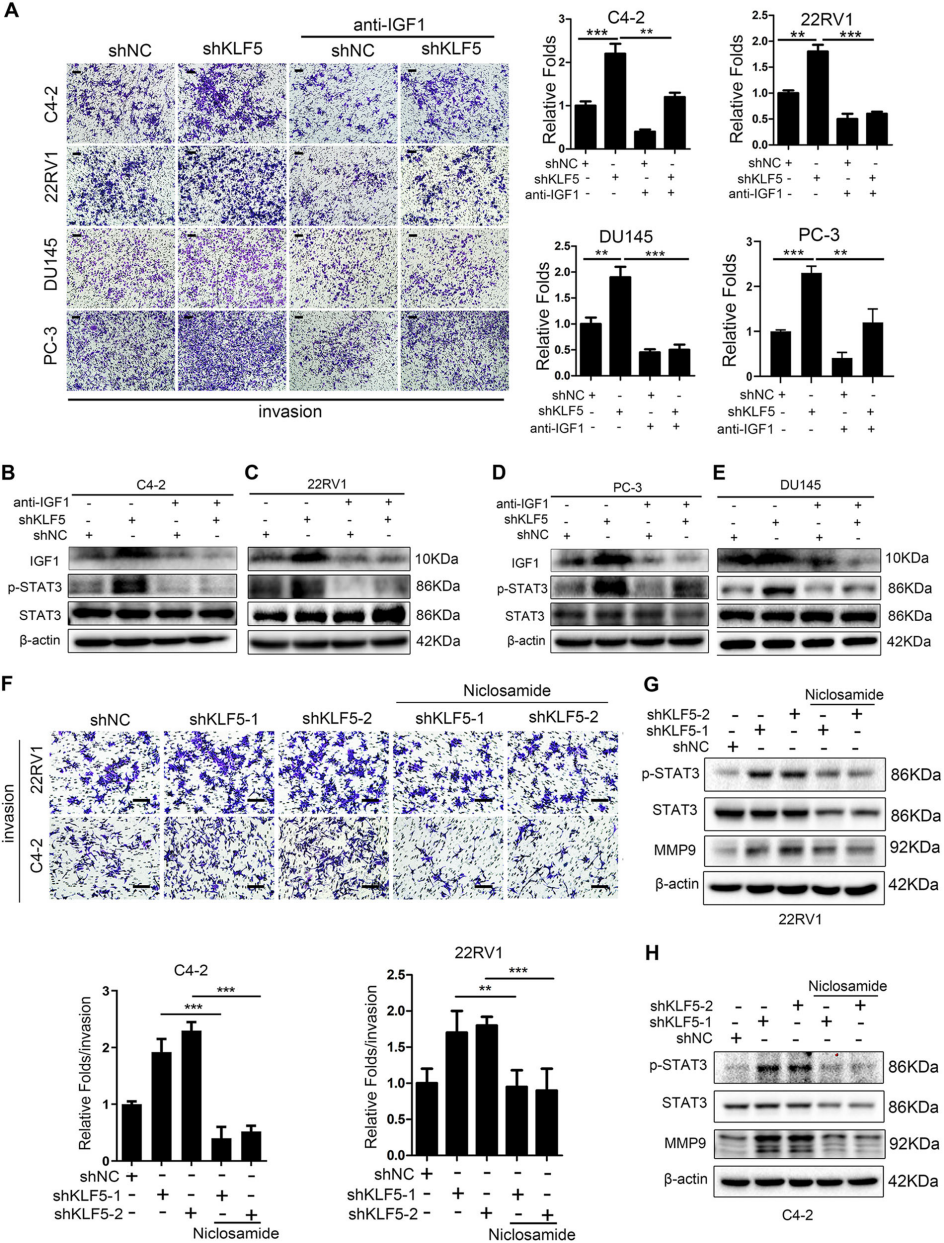
Blocking of IGF1/STAT3 pathway suppresses the cell invasive ability enhanced by KLF5 downregulation. **a** Representative Transwell invasion photographs and quantification of invasive abilities of shKLF5 subtypes of C4-2, 22RV1, PC-3, and DU145 cells treated with neutralizing antibody of IGF1 for 24 h. **p <0.01, ***p <0.001 versus control. Scale bar = 100 μm. **b**–**e** Western blotting analysis of IGF1, p-STAT3, and STAT3 expression in C4-2, 22RV1, PC-3, and DU145 cells transfected with shKLF5 or shNC after treatment with neutralizing antibody of IGF1 for 24 h. **f** Representative pictures and quantification analysis of invasion assays in C4-2 and 22RV1 cells transfected with shKLF5 or shNC after treatment with STAT3 inhibitor niclosamide. Scale bar = 100 μm. Each experiment was repeated at least three times and the result of a representative experiment is shown. **p < 0.01, ***p < 0.001. 18S was used as an internal loading control. **g**, **h** Western blotting analysis of KLF5, p-STAT3, STAT3, and MMP9 expression levels in C4-2/shKLF5 (**g**) and 22RV1/shKLF5 (**h**) sublines after treatment with 0.5 μM of STAT3 inhibitor niclosamide for 24 h.

To further clarify the function of IGF1 in cell invasion promoted by downregulation of KLF5, we treated C4-2, 22RV1, DU145, and PC-3 cells with neutralizing antibody of IGF1. Blocking of IGF1 not only inhibited cell invasive ability in control cells but also significantly reduced the increase in the invasiveness of KLF5 knockdown cells (Fig. 5a), indicating that IGF1 plays an important role in cell invasion regulated by KLF5. Similarly, the effect of KLF5 knockdown on p-STAT3 activation was blocked by neutralizing antibody of IGF1 (Fig. 5b–e), confirming that KLF5 knockdown enhanced the activation of IGF1/STAT3 pathway. To further investigate whether knockdown of KLF5 increased the invasiveness of PCa cells by activating STAT3, we suppressed STAT3 activation with 0.5 μM STAT3 inhibitor niclosamide, and found that blocking STAT3 activation inhibited PCa cell invasion enhanced by KLF5 knockdown (Fig. 5f). Moreover, as shown in Fig. 5g, h, p-STAT3 and MMP9 protein levels were increased by KLF5 knockdown, while niclosamide treatment suppressed the expression of not only p-STAT3 but also MMP9, suggesting that activation of STAT3/MMP9 is essential for the increased cell invasive ability by KLF5 knockdown in PCa cells.

Thus, we found that blocking the IGF1/STAT3 pathway suppressed the enhanced cell invasive ability caused by KLF5 downregulation, indicating the pivotal role of the IGF1/STAT3 signaling pathway in PCa cells.

### KLF5 binds cooperatively with HDAC1 on IGF1 promoter and suppresses its transcription

**Fig. 6:**
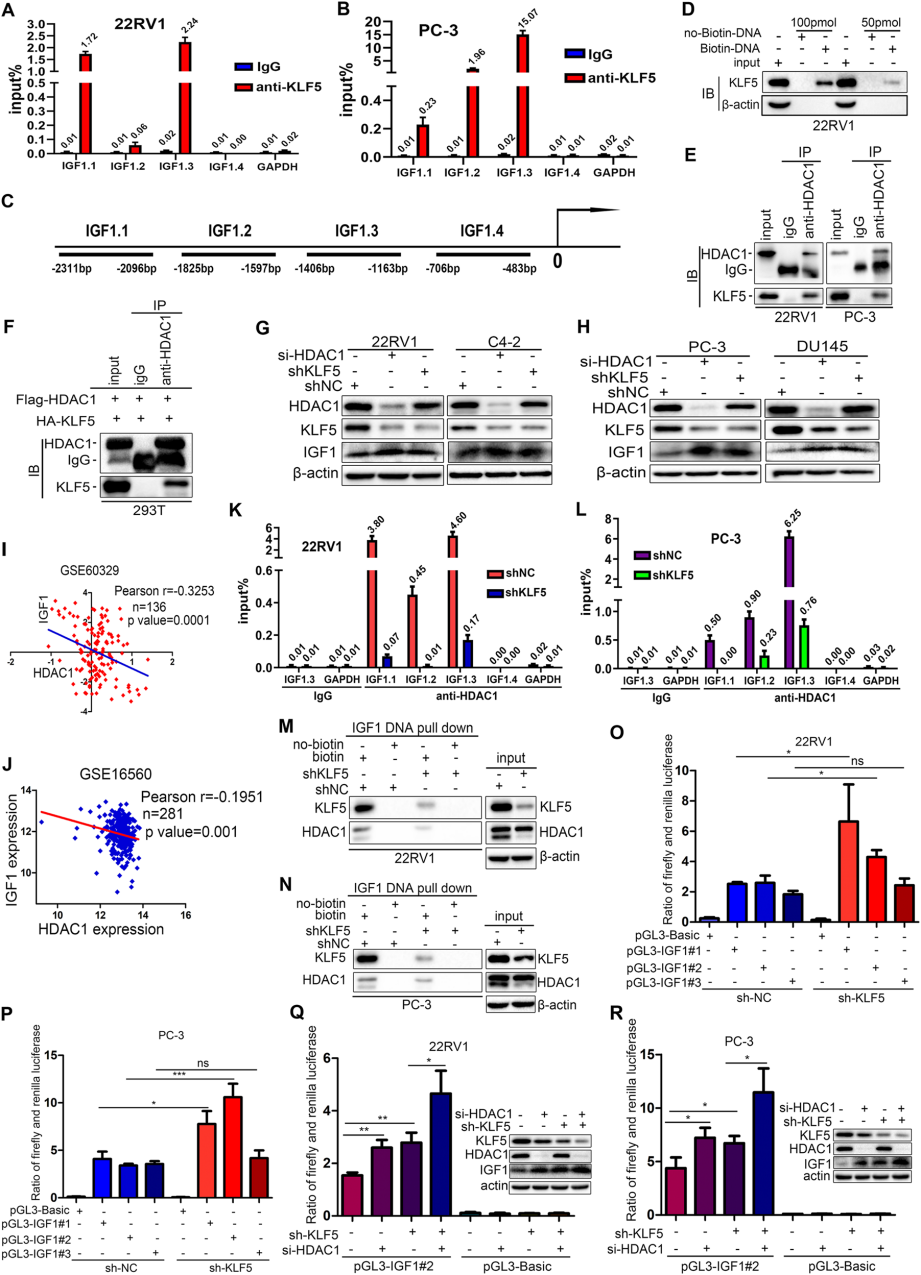
KLF5 binds on the *IGF1* promoter cooperatively with HDAC1 and suppresses the transcription of IGF1. **a**, **b** Chromatin immunoprecipitation (ChIP) assay of binding sites of KLF5 on the *IGF1* promoter detected by qPCR in 22RV1 and PC-3 cells. *GADPH* promoter was served as a negative control. **c** The map of IGF1 promoter, which indicates the regions analyzed in ChIP assay. **d** Oligonucleotide DNA pull-down assay was performed to verify that the DNA fragment amplified by PCR could pull-down KLF5 protein in 22RV1 cells; the pull-down protein was detected by western blotting. **e**, **f** Co-immunoprecipitation (Co-IP) assay of the mutual interaction of KLF5 and HDAC1 proteins at endogenous (22RV1 and PC-3) (**e**) and exogenous levels (293T) (**f**), as detected by western blotting analysis. IgG was used as the negative control, while input was a positive control. **g**, **h** Western blotting analysis of HDAC1, KLF5, and IGF1 protein expression in 22RV1, C4-2, PC-3, and DU145 cells transfected with shKLF5 lentivirus and si-HDAC1 siRNA. **i**, **j** Expression of IGF1 and HDAC1 mRNA was negatively correlated in prostate cancer tissues, as per analysis in GSE16560 and GSE60329. **k**, **l** ChIP-qPCR assay of HDAC1 binding on *IGF1* promoter in 22RV1 and PC-3 cells transfected with shKLF5 or shNC. Treatment with IgG was used as a negative control. **m**, **n** Oligonucleotide DNA pull-down assays were performed to verify that 100 pmol specific DNA fragment precipitated HDAC1 protein in NC and shKLF5 sublines of 22RV1 and PC-3 cells. No-biotin labeled DNA was used as a negative control. **o**, **p** Dual-glo luciferase assay indicated that knockdown of KLF5 in PC-3 and 22RV1 cells promoted the transcriptional activity of *IGF1* promoter. **q**, **r** Dual-glo luciferase assay showed that knockdown of HDAC1 further increased the transcriptional activity of the IGF1 promoter activated by knockdown of KLF5 in 22RV1 and PC-3 cells. ns *p* > 0.05; *p <0.05, **p <0.01, ***p <0.001. The pGL3-IGF1#1, pGL3-IGF1#2, and pGL3-IGF1#3 indicate three different oligonucleotides located in the proximal region of the IGF1 promoter. Plasmid pGL3-Basic served as a negative control. KLF5, HDAC1, and IGF1 expression in 22RV1 and PC-3 cells treated with shKLF5 and si-HDAC1 detected by western blotting.

KLF5 is a transcription factor that binds directly to specific motifs on the promoters of target genes. Since KLF5 could inhibit IGF1 expression at both mRNA and protein levels, we hypothesized that KLF5 might be an important regulator of IGF1 transcription. First, we determined whether KLF5 could bind to the *IGF1* promoter. CHIP-qPCR assay in PC-3 and 22RV1 cells showed that KLF5 could bind to IGF1.3 sequence on the *IGF1* promoter containing CA-box (CACCC) motif and GC-rich element (GGGCCC) (Fig. 6a–c). The oligo pull-down assay in 22RV1 cells also confirmed that KLF5 bound to IGF1.3 sequence of the *IGF1* promoter (Fig. 6d, Fig. S2, Supplementary methods).

HDAC1 is a member of histone deacetylases (HDACs), which remove acetyl groups from histones in the transcription complex and then inhibit gene transcription by tightening the chromatin^23^. To verify this, we employed a Co-IP assay and observed that exogenous and endogenous KLF5 could interact with HDAC1 in PC-3 and 22RV1 (Fig. 6e), and 293T cells (Fig. 6f). Furthermore, western blotting showed that both knockdowns of KLF5 and HDAC1 could upregulate IGF1 protein in C4-2, 22RV1, DU145, and PC-3 PCa cells (Fig. 6g, h). Interestingly, knockdown of HDAC1 decreased KLF5 expression, while KLF5 downregulation had little effect on HDAC1 expression. Notably, analysis from the GEO databases GSE16560 and GSE60329 showed that expression of HDAC1 mRNA was negatively correlated with IGF1 expression in PCa tissues (Fig. 6i, j), indicating that HDAC1 is a repressor of IGF1 expression.

Considering that both KLF5 and HDAC1 interact with each other and inhibit the expression of IGF1, we speculated that KLF5 could recruit HDAC1 to form a KLF5-HDAC1 protein complex, which can bind on *IGF1* promoter and suppress transcription of IGF1. To test the hypothesis, we employed CHIP-qPCR assay with the HDAC1 antibody and found that HDAC1 could bind to *IGF1.1*, *IGF1.2*, and *IGF1.3* regions on *IGF1* promoter, and this binding was decreased by KLF5 knockdown (Fig. 6k, l). Similarly, as shown in Fig. 6m, n, both KLF5 and HDAC1 could bind to IGF1.3 region of *IGF1* promoter, while KLF5 knockdown reduced the HDAC1 binding as detected by oligonucleotides pull-down assay and western blot analysis. Collectively, these results demonstrated that both KLF5 and HDAC1 bind to the *IGF1* promoter, and the binding of HDAC1 is dependent on KLF5.

To explore whether binding of KLF5 and HDAC1 on the *IGF1* promoter suppressed its transcription, we cloned three different DNA fragments of *IGF1* promoter, inserted them into pGL3-basic luciferase reporter plasmids, and performed dual-luciferase activity assay in PC-3 and 22RV1 cells. First, as shown in Fig. 6o, p, knockdown of KLF5 alone in both 22RV1 and PC-3 cells could significantly increase the transcriptional activity of the *IGF1* promoter containing CA-box and GC-rich element, which is consistent with results from the ChIP assay. Next, knockdowns of both KLF5 and HDAC1 markedly increased transcriptional activity of *IGF1* promoter and IGF1 protein expression as detected by dual-luciferase reporter assay and western blotting analysis. Also, the simultaneous knockdown of KLF5 and HDAC1 further enhanced the transcriptional activity of the *IGF1* promoter and IGF1 protein expression in both 22RV1 and PC-3 cells (Fig. 6q, r).

Taken together, these results showed that the cooperative binding of KLF5 with HDAC1 on the *IGF1* promoter suppressed its transcriptional activity and inhibited the transcription of IGF1.

## Discussion

KLF5 is considered a tumor suppressor in PCa as it is frequently deleted or downregulated. It has been reported that KLF5 suppressed PCa angiogenesis via attenuating AKT/HIF1α^24^, and inhibited cell proliferation through activation of FOXO1 transcription^6^. However, the association between KLF5 expression and the clinical characteristics of PCa is still not clear. To elucidate the role of KLF5 in PCa progression, we analyzed KLF5 expression in TCGA and GEO databases, as well as using PCa tissue microarray, and found KLF5 expression to be significantly decreased in PCa. The decrease at both mRNA and protein levels was accompanied by tumor progression. Also, KLF5 downregulation was associated with shorter survival of patients. Interestingly, KLF5 expression was lower in PCa metastases than in localized PCa tissues (Fig. 2a–c), indicating that KLF5 downregulation might be associated with PCa invasion and metastasis. This was also supported by the high frequency of deletion of KLF5 in PCa metastases^20^ (Fig. 1f, g) and the frequent loss of KLF5 in aggressive forms during the evolution of PCa^10^.

**Fig. 7:**
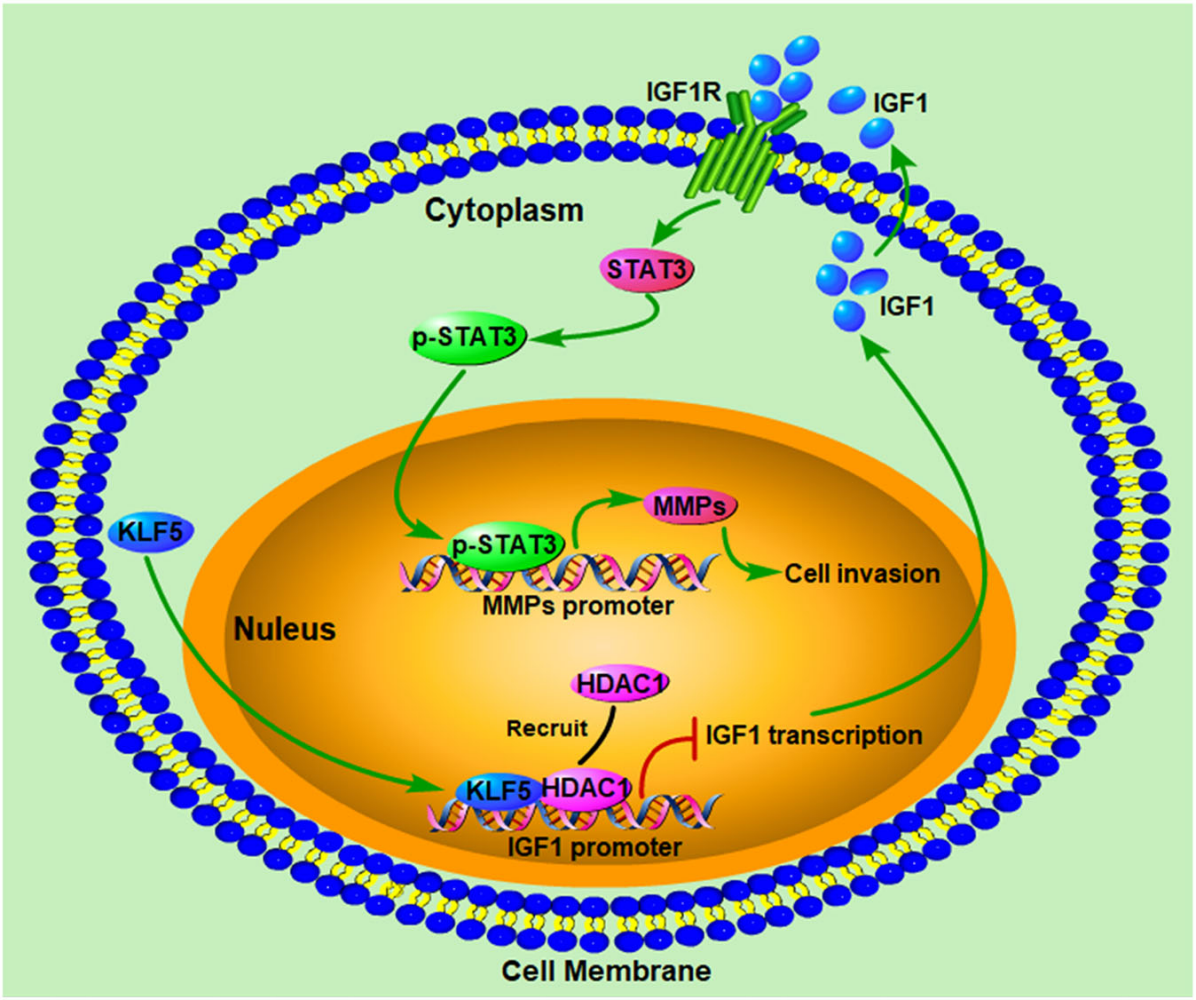
Schematic shows enhanced prostate cancer invasion by KLF5 downregulation. KLF5 and HDAC1 cooperatively bind on the promoter of IGF1 and suppress IGF1 transcription. KLF5 downregulation in prostate cancer leads to increased IGF1 expression. Subsequently, the autocrine IGF1 activates STAT3 and promotes the expression of its downstream genes, such as MMPs, enhancing the invasive ability of prostate cancer.

To assess the effect of KLF5 downregulation on PCa invasion, we knocked down KLF5 in PCa cells that resulted in increased invasive ability both in vitro and in vivo (Fig. 2). Furthermore, we found that KLF5 downregulation enhanced PCa invasion by activating the IGF1/STAT3 pathway (Figs. 3–5), and cooperative binding of KLF5 with HDAC1 on *IGF1* promoter suppressed the transcription of IGF1 (Fig. 6). Thus, our results indicated that KLF5 could be an important suppressor of tumor invasion and metastasis of PCa because KLF5 deletion/downregulation could promote the expression of a tumor cell autocrine cytokine and the subsequent cell signaling to enhance PCa cell invasive ability (Fig. 7).

IGF1 binds to IGF1 receptor (IGF1R) and insulin receptor, activating hallmark pathways associated with cancer^25^. It has been reported that circulating IGF1 is positively correlated with PCa risk^26^. Also, overexpression of IGF1 in the prostate epithelium in transgenic animals resulted in spontaneous neoplasia in the mouse prostate^27^, indicating that IGF1 is a promoter of PCa progression. In the present study, we found that knockdown of IGF1 and its blockage with antibody significantly suppressed the activation of STAT3 in PCa cells (Figs. 3 and 4), which is consistent with a previous study that IGF1/IGF1R could activate STAT3 and its downstream target genes such as MMP9^22^. Furthermore, knockdown of IGF1 decreased the invasive ability of PCa cells. These results indicated that IGF1 might promote PCa cell invasion through the activation of STAT3. On the other hand, blocking of IGF1 with its antibody attenuated the increase in STAT3 activity and invasive ability induced by KLF5 knockdown, thus identifying IGF1 as an important promoter of PCa invasion. KLF5 might inhibit cell invasion by suppressing the transcription of IGF1 in tumor cells and subsequently inhibiting its downstream activation of STAT3/MMP9.

HDACs play an important role in tumor development and progression by modifying histone and nonhistone proteins^28^. HDAC1, a member of Class I HDACs, exerts transcriptional repression function by regulating the balance of histone deacetylation and is present in several multiprotein repressor complexes^23^. However, in PCa, the function of HDAC1 in tumor progression is not clear. In the present study, we found that the expression of HDAC1 was negatively correlated with IGF1 expression and knockdown of HDAC1 upregulated IGF1 in PCa cells. Furthermore, HDAC1 bound on the *IGF1* promoter cooperatively with KLF5 to suppress the transcription of IGF1 as a co-repressor. These findings demonstrated the participation of HDAC1 in suppressing IGF1 expression in PCa and suggested that inhibition of HDAC1 might lead to increased STAT3 activity and cell invasive ability, and, therefore, this may be a concern for the potential application of HDAC inhibitors in PCa treatment.

In conclusion, our results indicate that downregulation of KLF5 induces activation of the IGF1/p-STAT3 signaling pathway, which might further promote the invasive ability of PCa cells. Thus, KLF5 appears to be an important repressor of cell invasion via regulation of the IGF1/p-STAT3 signaling pathway. Targeting downregulation of KLF5 and the ensuing activation of p-STAT3 might serve as potential targets toward precision medicine of PCa in the clinic.

## Supplementary information

Supplementary Materials and Methods

Supplementary Figure Legends

Supplemental Figure 1

Supplemental Figure 2
