# Supplementary material for: KLF5 inhibits STAT3 activity and tumor metastasis in prostate cancer by suppressing IGF1 transcription cooperatively with HDAC1
Source: Cell Death Dis. 2020 Jun 16;11(6):466. doi: 10.1038/s41419-020-2671-1 (PMC7297795; doi:10.1038/s41419-020-2671-1)
Supplement: Supplementary file 1 — Supplementary Materials and Methods [file 41419_2020_2671_MOESM1_ESM.docx]

**Supplementary Table 1. Primer sequence**

| primers | sequence |  |  |  |
| --- | --- | --- | --- | --- |
| KLF5-F  KLF5-R  18S-F  18S-R  MMP1-F | CAGAGGACCTGGTCCAGACAAG  GAGGCCAGTTCTCAGGTGAGTG  GCAATTATTCCCCATGAACG  GGCCTCACTAAACCATCCAA  GGGGCTTTGATGTACCCTAGC |  |  |  |
| MMP1-R | TGTCACACGCTTTTGGGGTTT |  |  |  |
| MMP2-F | GATACCCCTTTGACGGTAAGGA |  |  |  |
| MMP2-R | CCTTCTCCCAAGGTCCATAGC |  |  |  |
| MMP9-F  MMP9-R  MT1MMP-F  MT1MMP-R | AGACCTGGGCAGATTCCAAAC  CGGCAAGTCTTCCGAGTAGT  CATCTGTGACGGGAACTTTGA  GGCAGTGTTGATGGACGCA |  |  |  |
| MTA1-F | CATCAGAGGCCAACCTTTTCG |  |  |  |
| MTA1-R | GCACGTATCTGTCGGTGGTC |  |  |  |
| MET-F | AGCAATGGGGAGTGTAAAGAGG |  |  |  |
| MET-R | CCCAGTCTTGTACTCAGCAAC |  |  |  |
| IGF1-F | GCTCTTCAGTTCGTGTGTGGA |  |  |  |
| IGF1-R  EZH2-F  EZH2-R  FLT4-F  FLT4-R  HGF-F  HGF-R  HIF-2 alpha-F  HIF-2 alpha-R  NEDD9-F  NEDD9-R  SRC-F  SRC-R  beta-catenin-F  beta-catenin-R | GCCTCCTTAGATCACAGCTCC  AATCAGAGTACATGCGACTGAGA  GCTGTATCCTTCGCTGTTTCC  TGCACGAGGTACATGCCAAC  GCTGCTCAAAGTCTCTCACGAA  GCTATCGGGGTAAAGACCTACA  CGTAGCGTACCTCTGGATTGC  TTGCTCTGAAAACGAGTCCGA  GGTCACCACGGCAATGAAAC  ATGGCAAGGGCCTTATATGACA  TTCTGCTCTATGACGGTCAGG  TGGCAAGATCACCAGACGG  GGCACCTTTCGTGGTCTCAC  AAAGCGGCTGTTAGTCACTGG  CGAGTCATTGCATACTGTCCAT |  |  |  |

**Supplementary Table 2. siRNA/shRNA sequence**

| KLF5 shRNA  shKLF5-1: 5ʹ-CCTGAGTTCACCAGTATATTC-3ʹ  shKLF5-2: 5ʹ-CCTCAAATGACAGACCTAACT-3ʹ |
| --- |
| IGF1 siRNA  si-IGF1#1: 5’-CTGGTGG-ATGCTCTTCAGT-3’  si-IGF1#2: 5’-CTTCCGGAGCTGTGATCTA-3’  si-IGF1#3: 5’-AGGAGGCTGGAGATGTATT-3’ |
| si-HDAC1 (RIBOBIO)  5’-CTAATGA-GCTTCCATACAA-3’ |
|  |

**Supplementary Table 3. Region-specific primers for ChIP-qPCR assay**

| primers | sequence |  |  |  |
| --- | --- | --- | --- | --- |
| IGF1.1-F  IGF1.1-R  IGF1.2-F  IGF1.2-R  IGF1.3-F | 5’-TCGCTGGAGTACAGCATCTG-3’  5’-CATTACAGGTGAAG-GCCAGG-3’  5’-AGGGATTACTCACACATCTG-3’  5’-GACACATTCTCTG-GGAAGAC-3’ 5’-ACATAGTGCACCATTGACAC-3’ |  |  |  |
| IGF1.3-R | 5’-CTGACACA-TCAACTGAAAAC-3’ |  |  |  |
| IGF1.4-F | 5’-CACAGGTTTGAGT-TATATGG-3’ |  |  |  |
| IGF1.4-R  GAPDH-p-F  GAPDH-p-R | 5’-CATTG-GATTGGTCCCTTTAG-3’  5'-CGGCTACTAGCGGTTTTACG-3'  5'-AAGAAGATGCGGCTGACTGT-3' |  |  |  |

**Supplementary Methods**

**Bioinformatics analysis**

We downloaded data from TCGA and GEO databases (GSE55945, GSE16560, GSE35988, GSE6919, and GSE60329) and analyzed KLF5 mRNA expression in normal prostate tissues, prostate cancer, and metastatic tissues. We analyzed KLF5 deletion by using the cBioPortal database (<http://www.cbioportal.org/>). GSE16560 dataset and GEPIA2 web (<http://gepia2.cancer-pku.cn/#index>) were used to analyze the relationship between KLF5 and prognosis of patients with prostate cancer. Notably, HCMDB (Human Cancer Metastasis Database) (<https://hcmdb.i-sanger.com/>) is an integrated database designed to store and analyze large scale expression data of cancer metastasis, which was used to analyze the role of KLF5 in different prostate cancer metastases. To analyze the linear correlation between different indicators, we downloaded GSE10560 and GSE60329 datasets from the NCBI-GEO database.

**RNA sequencing assay**

The transcriptome sequencing assay was performed in Novogene Bioinformatics Institute (Novogene Co., LTD, Beijing, China). In brief, 3 μg RNA per sample was used for RNA sample preparation. Then Sequencing libraries were generated by using the NEBNext® Ultra™ RNA Library Prep Kit for Illumina® (NEB, USA) following the manufacturer's recommendations. Then differential expression analysis of two conditions (C4-2-NC/C4-2 shKLF5) was performed by using the R package.

**Cell invasion assay**

Cell invasion was tested by Boyden chamber assay, obtained from Millipore (Schaffhausen, Switzerland). For the invasion assay, the upper chamber was coated with Matrigel (Sigma, St. Louis, MO, USA) and incubated in 37 °C with 5% CO_2_ for 2-4h, and then C4-2 (1×10^5^ cells), 22RV1 (1.2×10^5^ cells), 8×10^4^ cells of DU145 and PC-3 were seeded onto the upper chamber (8μm pore polycarbonate membrane filters) in 0.2 ml serum-free RPMI-1640. The plates were incubated for 36 h and then the upper surface of the chambers were wiped gently with a Q-tip and fixed with 4% Paraformaldehyde for 15 min, then stained with crystal violet for 8min followed by washing three times with PBS. The dyed cells were counted using an inverted microscope, and five random visions were taken, and the average number of cells were analyzed.

**Real-time quantitative PCR (RT-qPCR) assay**

Cells were harvested to extract total RNA with a fasten 2000 RNA extract kit following the manufacturer’s protocol, and total RNA was quantitated by absorbance at 260 nm. RNA (1500ng) was reverse-transcribed to cDNA using the PrimeScript™ RT reagent kit (Takara, Dalian, China) and quantitative PCR was performed with SYBR-Green PCR Master Mix (Takara) with the gene-specific primers. Primer sequences are listed in Supplementary Table 1. 18S was used as a loading control, and relative gene expression was calculated by the 2^−ΔΔCt^ method.

**Co-immunoprecipitation (Co-IP) assay**

For exogenous Co-IP experiment, HA-KLF5 plasmid and pCMV3- HDAC1-Flag (Sino Biological, Beijing, China) were co-transfected into 293T cells for 48 h. After treatment, cells were harvested and lysed using cell lysis buffer (50 mM Tris-HCl, pH 7.5, 150 mM NaCl, 1% Nonidet P-40, 0.5% sodium deoxycholate, and 1% protease inhibitor cocktail). Next, cell lysates were centrifuged, and the supernatants were incubated with HDAC1 antibody or normal mouse IgG (Beyotime Biotechnology, Shanghai, China) at 4°C overnight. Each sample received 20ul Dynabeads^TM^ linked -protein G (Thermo Fisher Scientific, Inc.) for 1 h at 4°C. The beads were washed five times with cell lysis buffer, and the precipitated proteins were further analyzed and detected by Western blotting. For endogenous Co-IP assay, 22RV1 and PC-3 cells were harvested and lysed using cell lysis buffer. The remaining steps and analyses were the same as the exogenous Co-IP experiment.

**Oligonucleotides DNA pull-down assay**

In brief, total genomic DNA was extracted from 22RV1 cells using the TIANamp Genomic DNA Kit (TIANGEN, Beijing, China). The specific DNA fragment was amplified by PCR with biotin-labeled or no label specific primers for IGF1.3 (F: 5’-ACATAGTGCACCATTGACAC-3, R: 5’-CTGACACATCAACTGAAA-AC-3’) from Sangon Biotech (Shanghai, China) and purified with E.Z.N.A. ® Cycle Pure Kit (OMEGA, Madison, WI, USA). Cells were lysed in lysis buffer (50mM Tris-HCl, pH7.4, with 150mM NaCl, 1mM EDTA, and 1%triton X-100) containing phosphatase inhibitors cocktail. Purified PCR products (50pmol or 100pmol) were added to cell lysates and rocked at 4°C for 16h. Subsequently, DNA-bound proteins were collected with 20μl Dynabeads^TM^ Myone^TM^ Streptavidin T1 (Invitrogen) at 4°C for 1 h, washed with lysis buffer for 4 times and identified by Western blotting.
