# Supplementary material for: KLF5 inhibits STAT3 activity and tumor metastasis in prostate cancer by suppressing IGF1 transcription cooperatively with HDAC1
Source: Cell Death Dis. 2020 Jun 16;11(6):466. doi: 10.1038/s41419-020-2671-1 (PMC7297795; doi:10.1038/s41419-020-2671-1)
Supplement: Supplementary file 2 — Supplementary Figure Legends [file 41419_2020_2671_MOESM2_ESM.docx]

**Supplemental Figure 1. (A)** Heatmap shows cells invasion-related gene profiling detected by microarray in conditional klf5 knock-out mouse dorsal prostates, as analyzed from GSE58719. (**B**) Effect of KLF5 overexpression on IGF1 mRNA expression in DU145 cells, as analyzed from GEO56343.

**Supplemental Figure 2.** (**A**) DNA gel electrophoresis analysis of DNA and PCR products for DNA pull-down in 22RV1 cells. (**B**) DNA gel electrophoresis analysis of DNA and PCR products in 22RV1and PC-3 cells, which was used to further perform oligo pull down assay.
