# Supplementary figures and images for: KLF5 inhibits STAT3 activity and tumor metastasis in prostate cancer by suppressing IGF1 transcription cooperatively with HDAC1
Source: Cell Death Dis. 2020 Jun 16;11(6):466. doi: 10.1038/s41419-020-2671-1 (PMC7297795; doi:10.1038/s41419-020-2671-1)

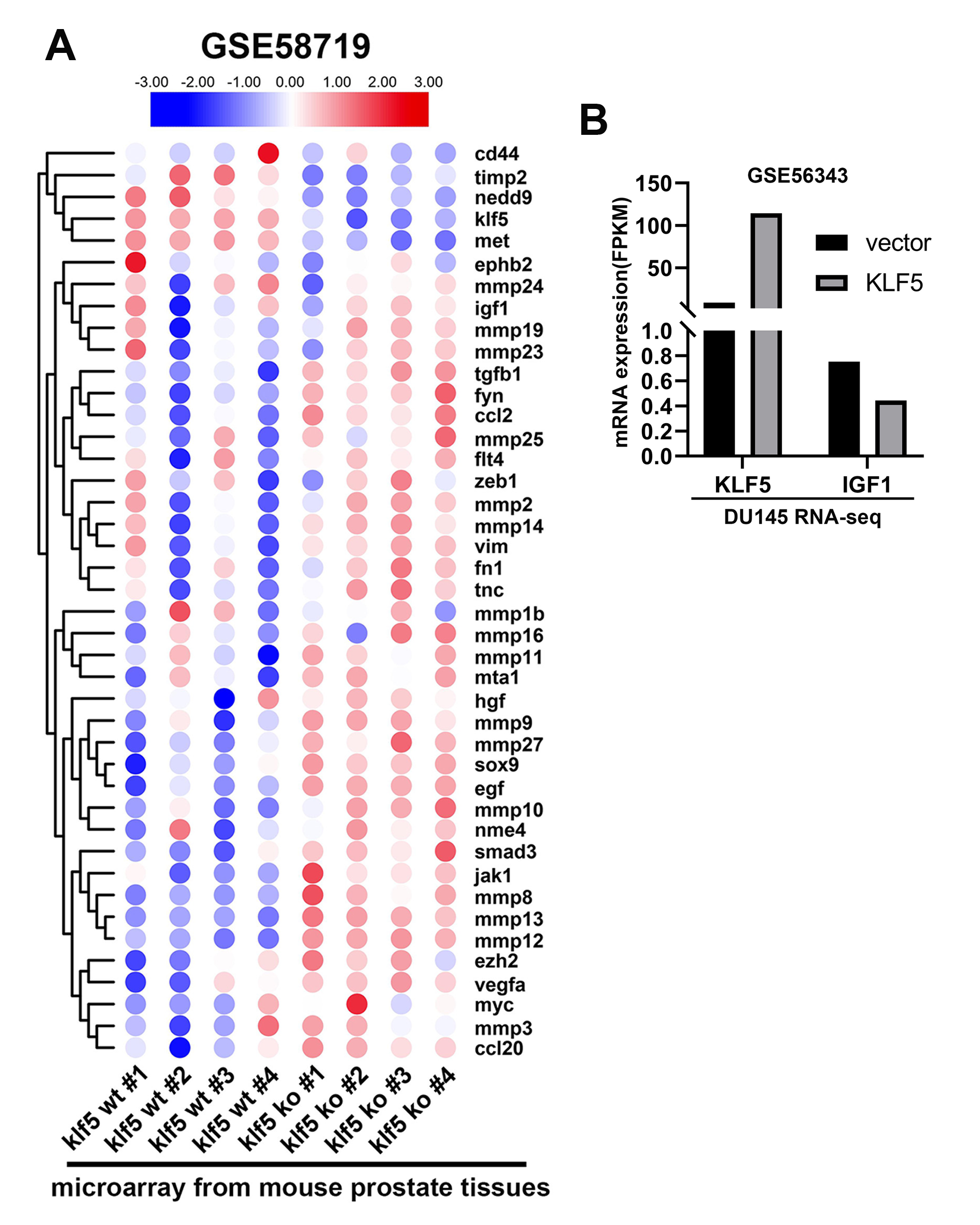

Supplement: Supplementary file 3 — Supplemental Figure 1 [file 41419_2020_2671_MOESM3_ESM.tif]
